# Epidemiology of Emergency Department Visits Before, During, and After the COVID-19 Pandemic: Experience From a Referral Center in Southeastern Brazil

**DOI:** 10.7759/cureus.102851

**Published:** 2026-02-02

**Authors:** Luis Gustavo Sedenho-Prado, Louise Buonalumi Tacito Yugar, Cleide Aparecida Moreira Silva, Andrea de Melo Alexandre Fraga, Luiz Roberto Lopes

**Affiliations:** 1 School of Medical Sciences, University of Campinas (UNICAMP), Campinas, BRA; 2 Department of Statistics, School of Medical Sciences, University of Campinas (UNICAMP), Campinas, BRA; 3 Department of Pediatrics, School of Medical Sciences, University of Campinas (UNICAMP), Campinas, BRA; 4 Department of Surgery, School of Medical Sciences, University of Campinas (UNICAMP), Campinas, BRA

**Keywords:** coronavirus, covid-19, emergency department, emergency visits, public health

## Abstract

Background

The COVID-19 pandemic and its related restrictions were associated with changes in emergency department (ED) utilization, with reductions reported in many regions worldwide. In Brazil, where EDs often serve non-urgent cases, the long-term impact remains unclear. Therefore, this study evaluated ED utilization trends across pre-pandemic, pandemic, and post-pandemic periods in a large tertiary hospital.

Methods

This retrospective observational study analyzed all ED visits recorded at the University of Campinas Clinics Hospital, a tertiary care referral hospital in southeastern Brazil, from January 2018 to December 2024. Data from the electronic medical record system were categorized into pre-pandemic, pandemic, and post-pandemic periods. Temporal trends in total, adult, and pediatric ED visits, as well as by medical specialty, were evaluated using joinpoint regression (Joinpoint Regression Program, version 4.9.0.1).

Results

Between Jan/18-Feb/20 (pre-pandemic), Mar/20-May/23 (pandemic), and Jun/23-Dec/24 (post-pandemic), 388,733 ED visits were recorded, including 329,111 adults and 59,622 pediatrics. Total visits declined by 39.5% from 2019 (69,519 visits) to 2020 (41,991), with reductions of 34.8% among adults (56,813 to 37,013) and 60.8% among children (12,706 to 4,978). Joinpoint analysis for total visits identified a sharp negative trend at the onset of the pandemic (p = 0.03), followed by a gradual recovery that did not reach pre-pandemic levels by 2024 (77.8% of baseline; pre-pandemic mean = 70,701; visits in 2024 = 54,987). Temporal patterns varied by specialty: neurology and some surgical specialties showed growth, while internal medicine, psychiatry, and ophthalmology exhibited distinct fluctuations and incomplete recovery.

Conclusion

The COVID-19 pandemic caused a marked and prolonged disruption in ED services utilization. Although visit volumes gradually recovered, they remained below pre-pandemic levels, with variable patterns across age groups and specialties. These findings highlight lasting shifts in healthcare-seeking behavior and the need for adaptive planning for future health crises.

## Introduction

SARS-CoV-2, the etiologic agent of COVID-19, can cause severe respiratory and systemic inflammation, particularly among patients with chronic comorbidities [1-3]. In February 2020, the first COVID-19 case was confirmed in Brazil, and one month later, the World Health Organization (WHO) declared a global pandemic. In response, governments worldwide implemented mitigation measures, such as social distancing, quarantine, and lockdowns, that profoundly affected healthcare utilization. Many regions subsequently reported substantial reductions in non-COVID-19 hospital admissions and emergency department (ED) visits, along with a parallel rise in telemedicine use [4-11].

In Brazil, where patients often rely on emergency services even for non-urgent conditions, these behavioral shifts may have been especially pronounced [12,13]. Previous national and regional reports suggested a sharp decline in emergency visits during the first pandemic waves [14-16]. However, few studies have evaluated whether this effect persisted after the WHO declared the end of the global public health emergency in May 2023. Furthermore, data from large tertiary care referral centers serving diverse urban populations remain limited.

The University of Campinas Clinics Hospital (HC-UNICAMP) is a tertiary care public hospital that provides care to approximately 6.5 million inhabitants in southeastern Brazil [17]. By the time the WHO declared the end of the COVID-19 global emergency in 2023, Brazil had already registered more than 38 million cases and 700 thousand deaths [18]. Understanding how ED utilization evolved across the pre-pandemic, pandemic, and post-pandemic periods is essential for assessing the indirect effects of COVID-19 and for strengthening preparedness for future health system disruptions. However, evidence describing long-term patterns in ED use throughout the full course of the pandemic and its aftermath remains limited.

Accordingly, we conducted a retrospective time-series analysis of ED visits at HC-UNICAMP from January 2018 through December 2024, using joinpoint regression to identify statistically significant changes in temporal trends. Unlike most prior studies that focused on the early pandemic period, this analysis includes the full pandemic timeframe and an extended post-pandemic follow-up. The primary objective was to evaluate temporal trends in total, adult, and pediatric ED visits, while the secondary objective was to examine specialty-specific patterns of ED utilization across the pre-pandemic, pandemic, and post-pandemic periods. This approach allows the assessment of whether ED utilization fully recovered or resulted in sustained changes in care-seeking behavior despite the resolution of acute pandemic restrictions. 

## Materials and methods

Study design and ethical approval

This single-center, retrospective observational study was conducted at the HC-UNICAMP, a tertiary care academic hospital that serves as the referral center for a macro-regional population of approximately 6.5 million people in southeastern Brazil. The study was approved by the Ethics Committee of the University of Campinas (IRB no. 5.479.286; June 21, 2022) and was conducted in accordance with the Declaration of Helsinki. The requirement for informed consent was waived owing to the retrospective nature of the study.

Data source and study population

Data were obtained from the hospital’s electronic medical record system, which prospectively captures all ED encounters at HC-UNICAMP. ED utilization data are routinely aggregated and exported on a monthly basis by the hospital’s informatics team and made available through the institutional intranet as structured tabular reports. These reports include, for each calendar month, the total number of ED visits and corresponding stratifications by patient population (adult or pediatric), medical specialty, and COVID-19-related status, as defined by institutional diagnostic coding and care pathways.

For the present study, we accessed the institutional intranet, gathered all tabular reports, and extracted monthly ED visit data covering the period from January 1, 2018, through December 31, 2024. All recorded ED visits during this interval were screened for inclusion. Visits primarily related to COVID-19 were excluded to allow focused analysis of non-COVID emergency care utilization. No additional exclusion criteria were applied.

For temporal analyses, ED visits were categorized into three predefined periods reflecting the evolution of the COVID-19 pandemic in Brazil, consistent with WHO declarations: pre-pandemic (January 2018 to February 2020), pandemic (March 2020 to May 2023), and post-pandemic (June 2023 to December 2024).

Outcomes

The primary endpoint was the monthly number of ED visits, stratified by patient group (adult, pediatric, and total). The secondary endpoint was the monthly number of ED visits by medical specialty. The age cutoff between adult and pediatric patients was 14 years, according to the HC-UNICAMP institutional classification. ED specialties include internal medicine, neurology, psychiatry, ophthalmology, trauma and emergency surgery, neurosurgery, and orthopedic surgery.

Statistical analysis

Monthly ED visit counts were organized in a structured spreadsheet for data management and descriptive analyses. Mean monthly visit volumes were calculated for the pre-pandemic and post-pandemic periods to allow descriptive comparison across time periods.

Temporal trends were formally analyzed using joinpoint regression using the Joinpoint Regression Program, Version 4.9.0.1 [19]. This method identifies statistically significant inflection points (“joinpoints”) where trends change direction or slope over time. Models were fitted to monthly visit counts, and the maximum number of joinpoints was selected according to program defaults. Statistical significance was assessed using two-sided tests with an α level of 0.05.

## Results

Study sample

The data included 388,733 ED visits between January 2018 and December 2024, which are presented in Table 1. Of these, 59,622 were from pediatric patients (15.3%), while 329,111 were from adult patients (84.7%), as demonstrated in Tables 2-3, respectively. In total, 52.5% were male (204,084) and 47.5% were female (184,649). Also, 72.72% were White (282,686), 21% were mixed-race Black (81,633), 5.79% were Black (22,507), 0.25% were Asian (971), 0.01% were Indigenous (42), and 0.23% did not inform their race (894). With the onset of the COVID-19 pandemic, visits declined in each category of the primary endpoint. In comparison to 2019, the total number of visits in 2020 dropped from 69,519 to 41,991, which represents a 39.5% reduction. The adult population visited our ED less frequently as well, with a reduction of 34.8% (56,813 visits in 2019 became 37,013 in 2020). Meanwhile, the pediatric cohort suffered the largest effect and presented 60.8% less visits, going from 12,706 in 2019 to 4,978 in 2020. Just after the first months of the pandemic, the monthly number of visits started to gradually increase in all categories of the primary endpoint, although they never recovered to the pre-pandemic levels. By the end of 2024, adult, pediatric, and total visits were 76.9% (45,105 vs. 58,596), 81.6% (9,882 vs. 12,102), and 77.8% (54,987 vs. 70,701) of the pre-pandemic mean registered before 2020, respectively.

**Table 1:** Total number of visits according to the month and year

All visits	January	February	March	April	May	June	July	August	September	October	November	December	Total
2018	6154	5538	6482	6226	6093	5638	5690	5818	5790	6623	6137	5694	71883
2019	6109	5414	6190	6490	6651	5569	5146	5563	5667	5933	5508	5279	69519
2020	5345	4805	3882	2111	2254	2388	2637	3049	3522	4321	4092	3585	41991
2021	4076	3698	2735	3091	3672	3423	3999	4806	4704	4146	4351	4288	46989
2022	3740	3839	4530	4431	4220	3398	3607	3552	3962	4424	4493	4352	48548
2023	4439	4335	5242	4344	4767	4445	4400	4644	4634	4734	4480	4352	54816
2024	4574	4543	4784	4944	4663	4531	4348	4596	4517	4709	4552	4226	54987

**Table 2:** Number of pediatric visits according to the month and year

Pediatric visits	January	February	March	April	May	June	July	August	September	October	November	December	Total
2018	778	881	1304	1351	1409	1137	871	900	679	309	870	1015	11504
2019	816	864	1210	1313	1232	1198	773	1052	1033	1225	1040	950	12706
2020	843	854	709	220	233	230	221	275	337	384	338	334	4978
2021	362	330	246	274	290	260	286	410	484	449	526	564	4481
2022	409	399	539	555	530	456	449	492	699	706	670	628	6532
2023	544	798	1223	877	964	791	622	761	772	804	732	651	9539
2024	556	673	868	1047	891	909	735	918	888	905	852	640	9882

**Table 3:** Number of adult visits according to the category of the month and year

Adult visits	January	February	March	April	May	June	July	August	September	October	November	December	Total
2018	5376	4657	5178	4875	4684	4501	4819	4918	5111	6314	5267	4679	60379
2019	5293	4550	4980	5177	5419	4371	4373	4511	4634	4708	4468	4329	56813
2020	4502	3951	3173	1891	2021	2158	2416	2774	3185	3937	3754	3251	37013
2021	3714	3368	2489	2817	3382	3163	3713	4396	4220	3697	3825	3724	42508
2022	3331	3440	3991	3876	3690	2942	3158	3060	3263	3718	3823	3724	42016
2023	3895	3537	4019	3467	3803	3654	3778	3883	3862	3930	3748	3701	45277
2024	4018	3870	3916	3897	3772	3622	3613	3678	3629	3804	3700	3586	45105

Total visits

**Figure 1:**
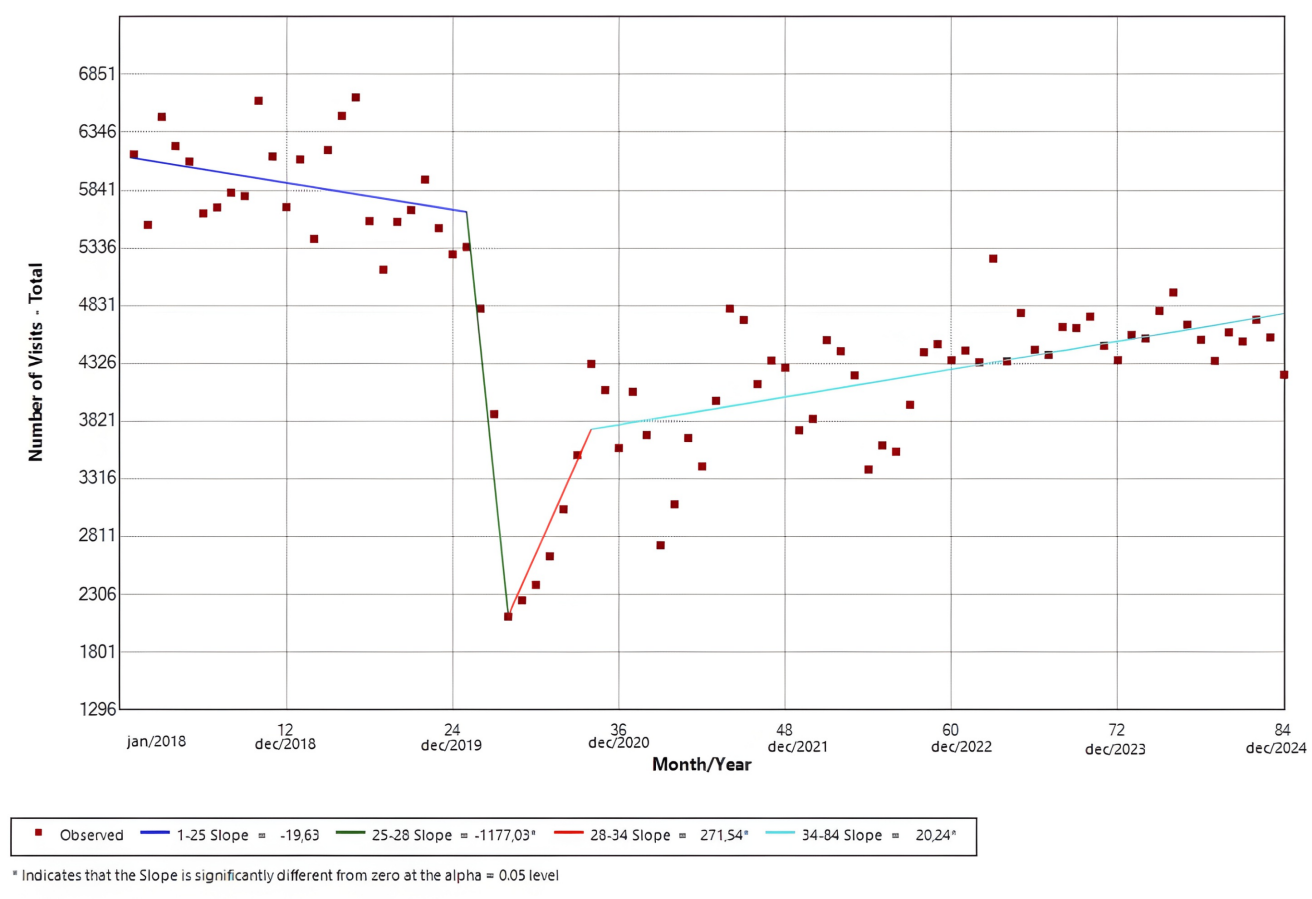
Joinpoint regression for all emergency room visits in the period

The software identified four distinct temporal trends. Slope 1 (Jan/18-Jan/20; p = 0.09) demonstrated a negative temporal trend that was not statistically significant. Slope 2 (Jan/20-Apr/20; p = 0.03) presented a statistically significant negative trend in the monthly visits. Compared to the mean number of pre-pandemic visits (5,830), the trough of slope 2 represented a reduction of 63.8% (Apr/20 = 2,111). In contrast, both slope 3 (Apr/20-Oct/20; p = 0.03) and slope 4 (Oct/20-Dec/24; p < 0.01) presented positive trends that were significant. The findings are shown in Figure 1.

Adult visits

**Figure 2:**
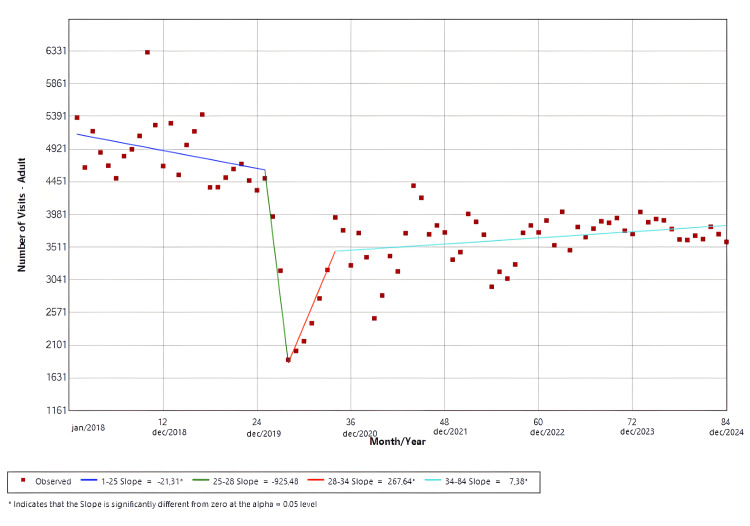
Joinpoint regression for adult visits in the emergency room

The software identified four distinct temporal trends. Slope 1 (Jan/18-Jan/20; p = 0.04) demonstrated a significant negative trend in the ED visits. Meanwhile, slope 3 (Apr/20-Oct/20; p = 0.02) and slope 4 (Oct/20-Dec/24; p = 0.04) presented positive trends that were significant. Slope 2 (Jan/20-Apr/20) demonstrated a negative trend, and its trough is 60.8% lower (Apr/20 = 1,891) than the pre-pandemic mean number of visits (4,830). However, this was not statistically significant (p = 0.07). The findings are shown in Figure 2.

Pediatric visits

**Figure 3:**
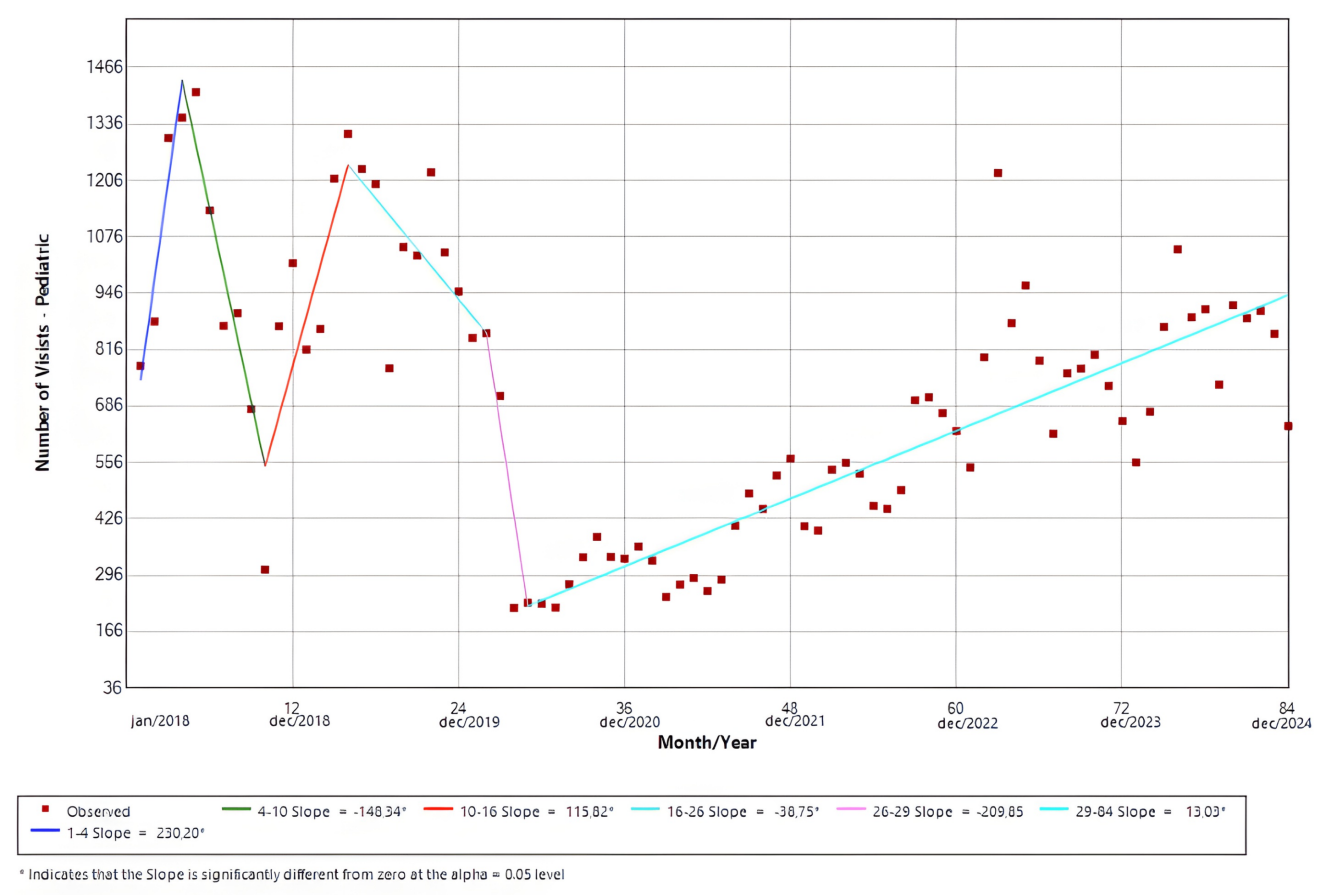
Joinpoint regression for pediatric visits in the emergency room

The software identified six distinct temporal trends. Slopes 1 (Jan/18-Apr/18; p = 0.01), 3 (Oct/18-Apr/19; p < 0.01), and 6 (May/20-Dec/24; p < 0.01) presented positive temporal trends that were statistically significant. Both slopes 2 (Apr/18-Oct/18; p < 0.01) and 4 (Apr/19-Feb/20; p = 0.02) demonstrated significant negative trends for the ED visits. Slope 5 (Feb/20-May/20; p = 0.26) was not statistically significant, despite its trough being 77.9% (Apr/20 = 220) lower compared to the mean pre-pandemic visits (996). The findings are shown in Figure 3.

Clinical specialties subanalysis

Internal Medicine

**Figure 4:**
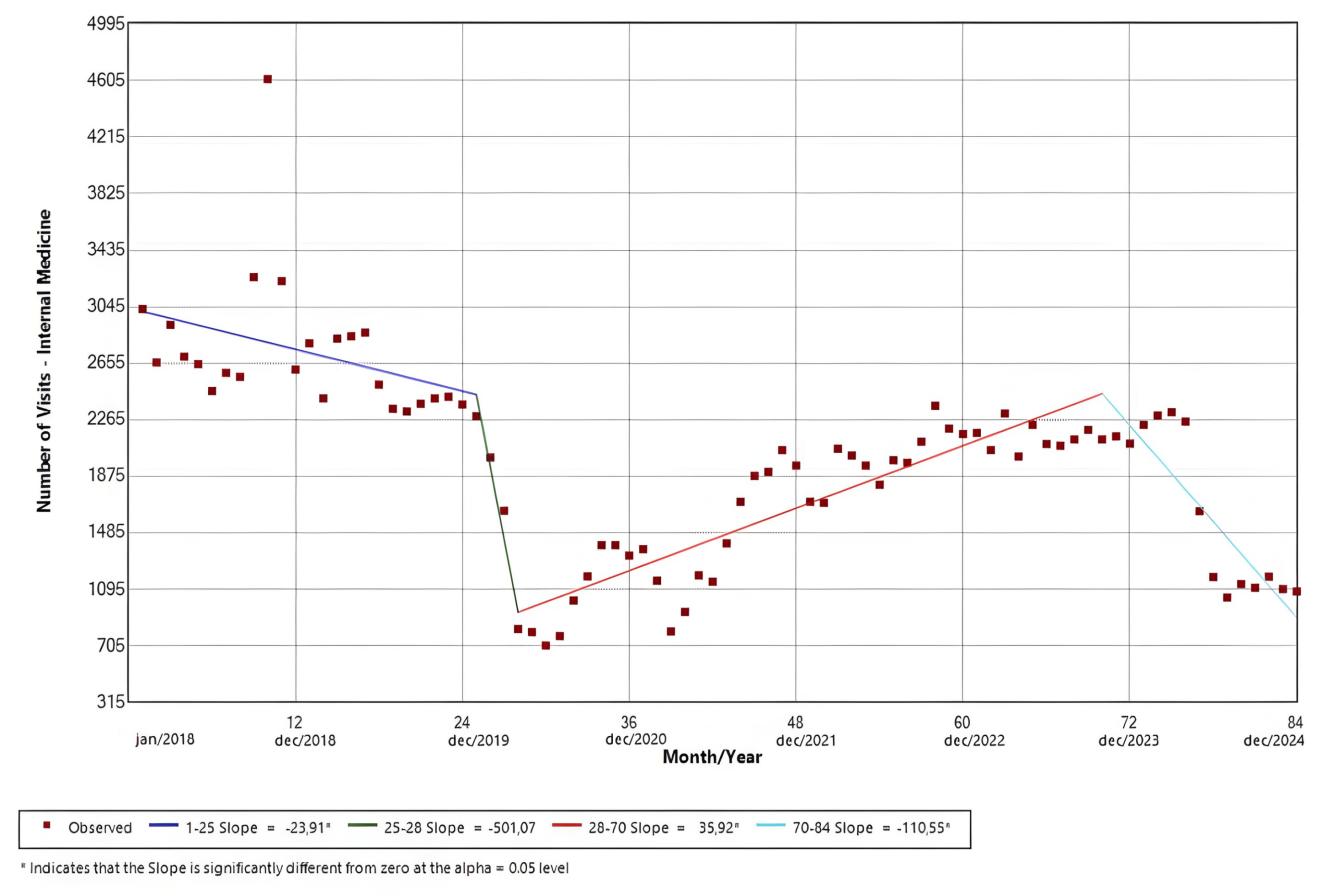
Joinpoint regression for clinical specialties-internal medicine

The software identified four distinct temporal trends. Slope 1 (Jan/18-Jan/20; p = 0.01) demonstrated a negative temporal trend that was statistically significant. Slope 2 (Jan/20-Apr/20; p = 0.27) presented a negative trend in the monthly visits that was not significant. In contrast, slope 3 (Apr/20-Oct/23; p < 0.01) presented a significant positive trend, and slope 4 (Oct/23-Dec/24; p < 0.01) demonstrated a significant negative trend. The findings are shown in Figure 4.

Neurology

**Figure 5:**
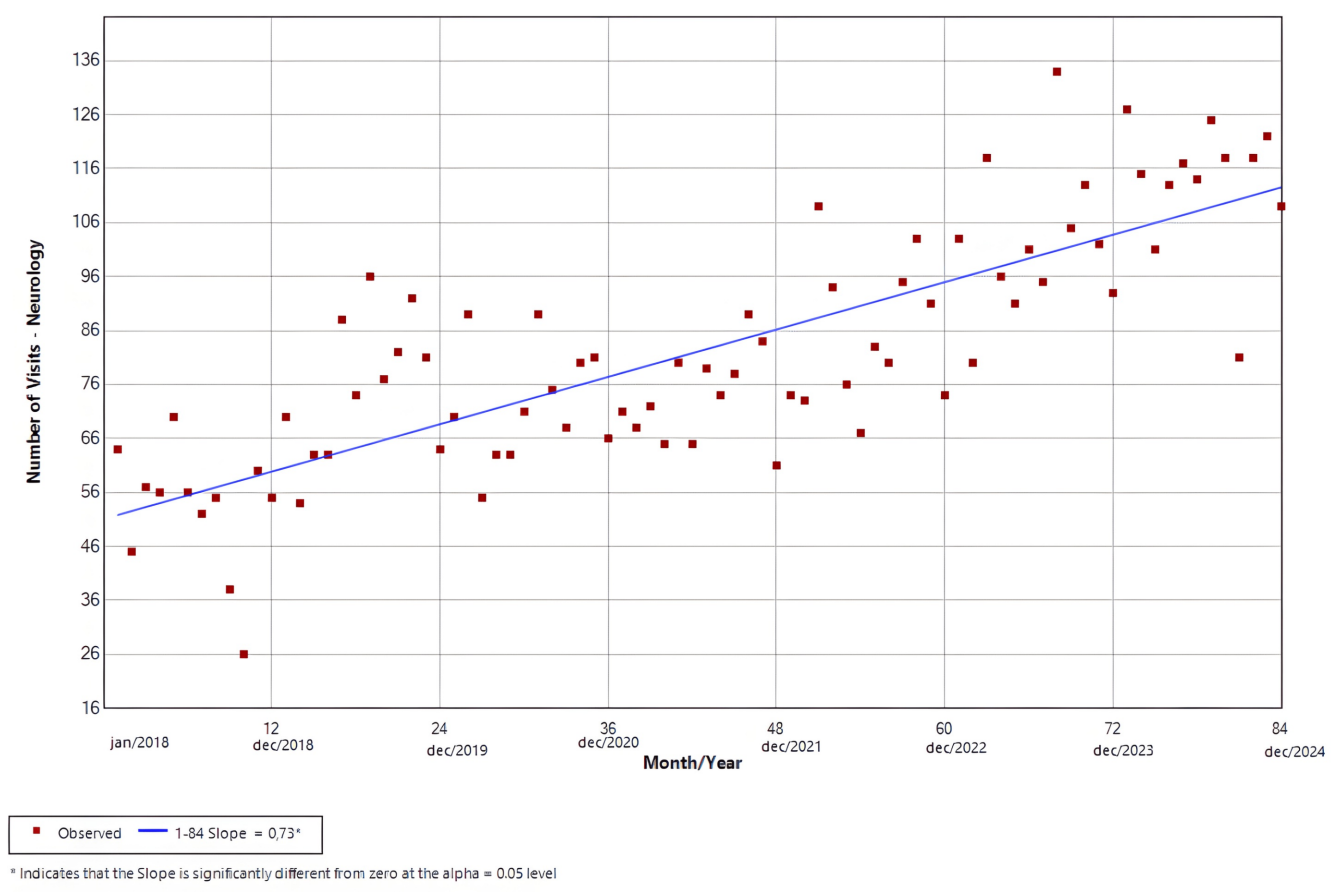
Joinpoint regression for clinical specialties-neurology

The software identified only one slope from January 2018 to December 2024, which had a positive trend and was statistically significant (p < 0.01). The findings are shown in Figure 5.

Psychiatry

**Figure 6:**
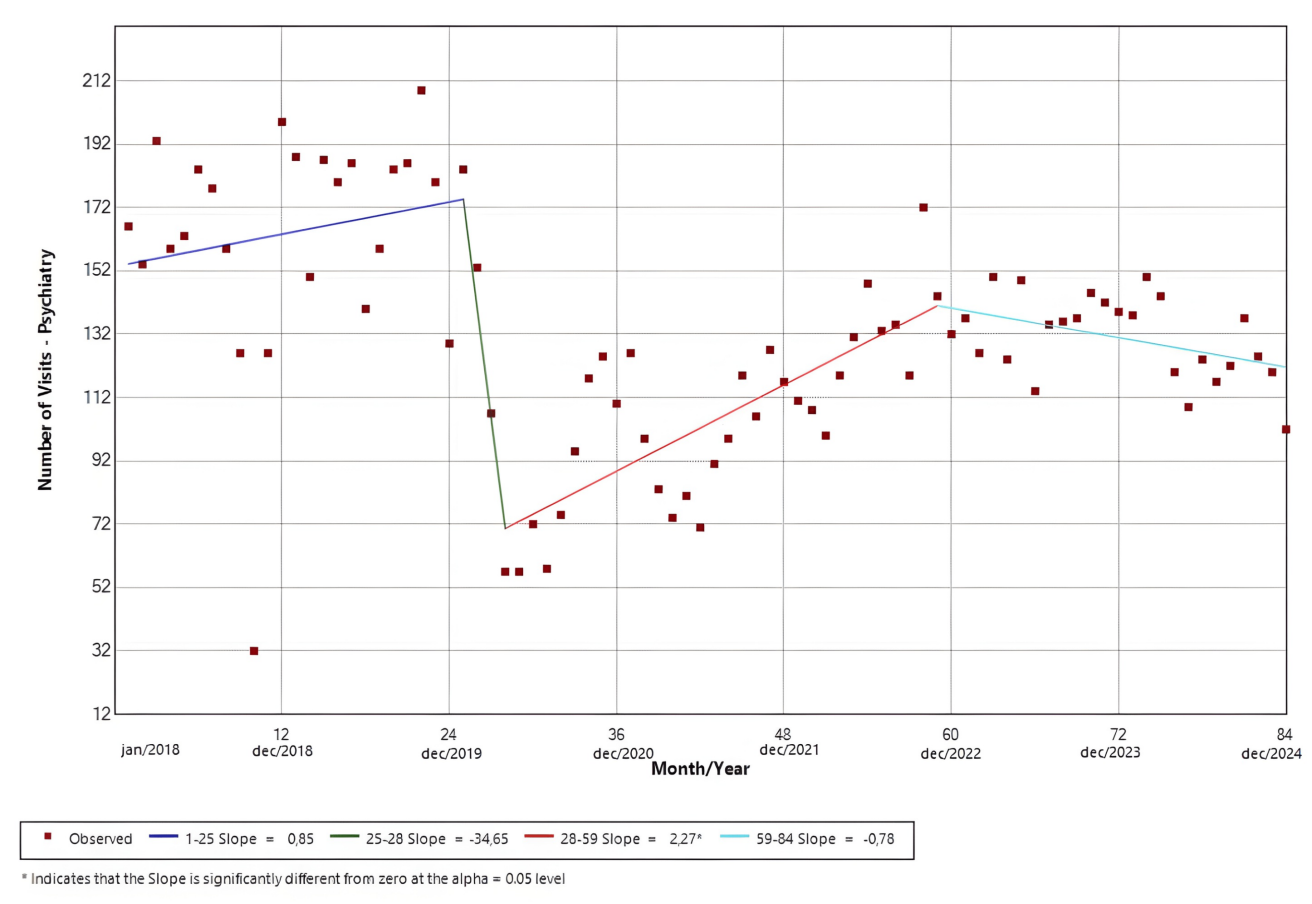
Joinpoint regression for clinical specialties-psychiatry

The software identified four distinct temporal trends. Slope 1 (Jan/18-Jan/20; p = 0.24) demonstrated a positive temporal trend, while slope 2 (Jan/20-Apr/20; p = 0.31) presented a negative trend in the visits. Slope 3 (Apr/20-Nov/22; p < 0.01) presented a positive trend, and slope 4 (Nov/22-Dec/24; p = 0.25) demonstrated a negative trend. Only the third slope had a statistically significant result. The findings are shown in Figure 6.

Ophthalmology

**Figure 7:**
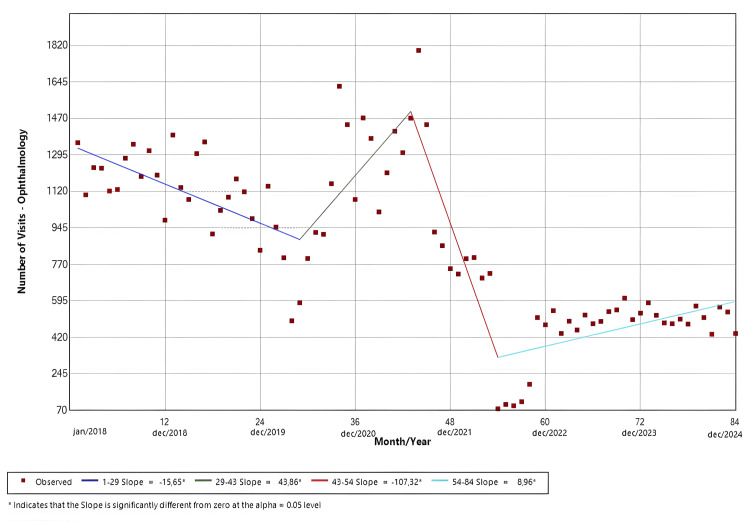
Joinpoint regression for clinical specialties-ophthalmology

The software identified four distinct temporal trends, which were all statistically significant. Slope 1 (Jan/18-May/20; p < 0.01) demonstrated a negative temporal trend, while slope 2 (May/20-Jul/21; p = 0.31) presented a positive trend in the ER visits. Afterwards, slope 3 (Jul/21-Jun/22; p < 0.01) presented a negative trend, and slope 4 (Jun/22-Dec/24; p = 0.01) demonstrated a positive trend. The findings are shown in Figure 7.

Surgical specialties subanalysis

Trauma and Emergency Surgery

**Figure 8:**
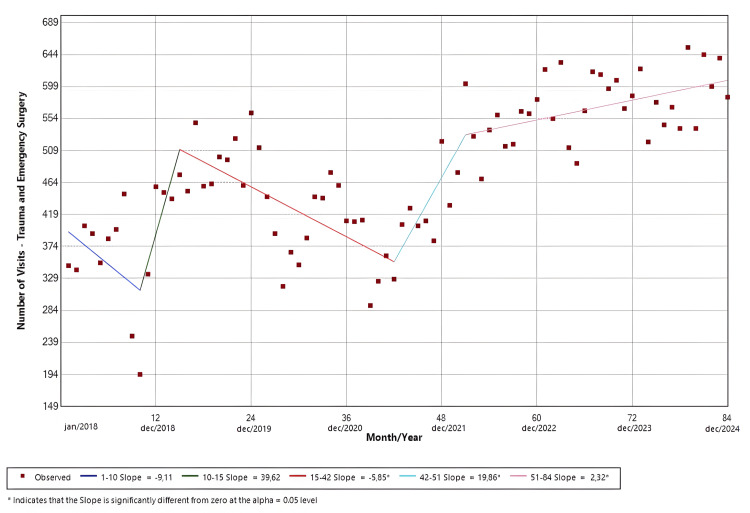
Joinpoint regression for surgical specialties-trauma and emergency surgery

The software identified five distinct temporal trends. Slopes 1 (Jan/18-Oct/18; p = 0.15) and 3 (Mar/19-Jun/21; p < 0.01) presented negative temporal trends, whereas slopes 2 (Oct/18-Mar/19; p = 0.07), 4 (Jun/21-Mar/22; p = 0.01), and 5 (Mar/22-Dec/24; p < 0.01) demonstrated positive trends in the ED visits. Only the results from slopes 3-5 were statistically significant. The findings are shown in Figure 8.

Neurosurgery

**Figure 9:**
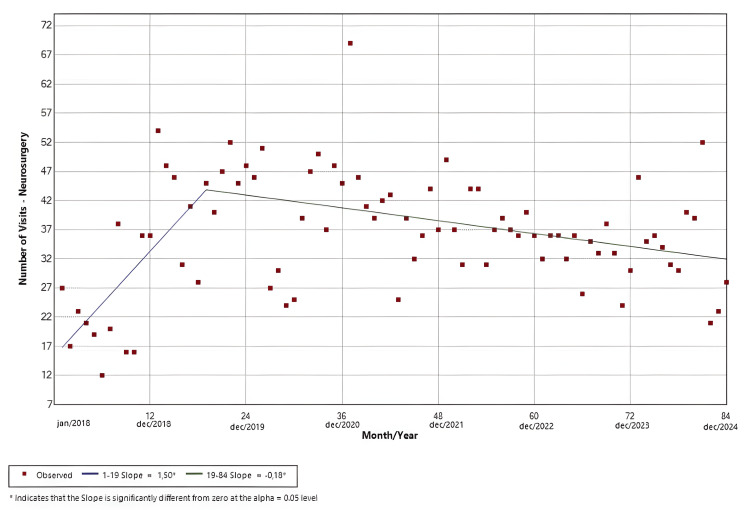
Joinpoint regression for surgical specialties-neurosurgery

The software identified two statistically significant slopes. The first slope (Jan/18-Jul/19; p < 0.01) had a positive trend, while the second (Jul/19-Dec/24; p < 0.01) had a negative trend. The findings are shown in Figure 9.

Orthopedics

**Figure 10:**
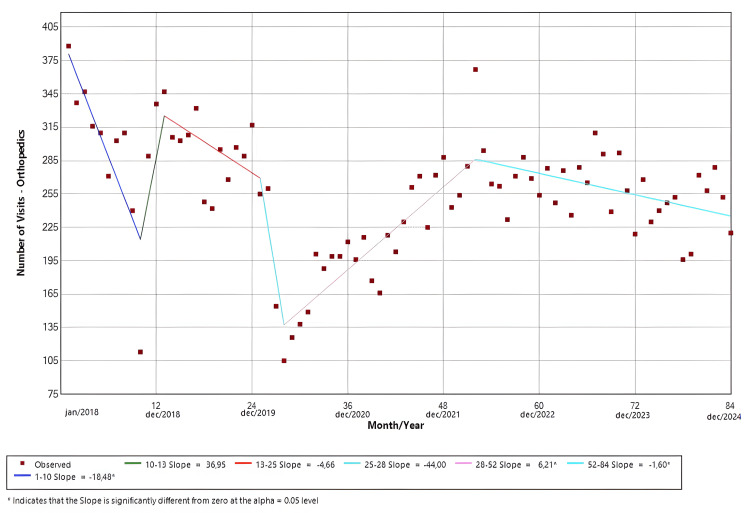
Joinpoint regression for surgical specialties-orthopedic surgery

The software identified six distinct temporal trends. Slopes 1 (Jan/18-Oct/18; p < 0.01), 3 (Jan/19-Jan/20; p = 0.054), 4 (Jan/20-Apr/20; p = 0.21), and 6 (Apr/22-Dec/24; p < 0.01) demonstrated negative temporal trends. Meanwhile, slopes 2 (Oct/18-Jan/19; p = 0.29) and 5 (Apr/20-Apr/22; p < 0.01) showed positive trends in the ED visits. Only the results from slopes 1, 5, and 6 were statistically significant. The findings are shown in Figure 10.

## Discussion

In this single-center time-series analysis spanning the pre-pandemic, pandemic, and post-pandemic periods, we identified a marked and sustained disruption in ED services utilization following the onset of the COVID-19 pandemic. Although sharp declines in ED visits during the early pandemic have been widely reported, our extended follow-up through December 2024 demonstrates that utilization did not fully return to pre-pandemic levels, with substantial heterogeneity across age groups and clinical specialties. By applying joinpoint regression over a prolonged timeframe, this study provides evidence that pandemic-related changes in emergency care-seeking behavior may persist well beyond the resolution of the acute public health emergency.

According to the Brazilian Ministry of Health, the COVID-19 outbreak in Brazil began in the state of São Paulo on February 26, 2020. By the time the WHO declared the end of the international emergency, nearly 7 million cases and more than 185,000 deaths had occurred in the state, making it the most affected region in the country [18]. The metropolitan area of Campinas accounted for over 600,000 cases and almost 10% of these deaths [18]. Given its high complexity and advanced diagnostic capabilities, HC-UNICAMP is the leading tertiary hospital in the region, providing high-quality, patient-centered care within the Brazilian public health system. However, the SARS-CoV-2 pandemic profoundly altered patterns of ED utilization in our hospital.

Before the pandemic, the HC-UNICAMP ED averaged approximately 5,900 visits per month. Following the implementation of social distancing measures, lockdowns, mobility restrictions, and healthcare reorganization policies under municipal decree Nº 20.771/2020 [20]; however, the number of visits dropped sharply, reaching a nadir approximately 64% below pre-pandemic levels (Apr/20 = 2,111; mean = 5,830). This decline was followed by a steep rebound through the end of 2020 and a more gradual increase thereafter; even so, overall utilization remained below baseline. Although the magnitude of decline observed in our institution exceeded that reported by many Brazilian, European, and North American centers, the overall pattern is consistent with prior studies describing substantial reductions in ED demand during the early pandemic phase, which documented declines ranging from 30% to 57% [4,15,21-23]. Our findings extend this literature by demonstrating that recovery may be incomplete even several years after the initial disruption.

Age-stratified analyses revealed divergent patterns between adult and pediatric populations. Adults comprised the majority of visits during the study period, and their ED utilization largely mirrored overall hospital trends. As shown in Figure 2, the nadir for this group represented a 60.8% reduction compared with the pre-pandemic average, although this difference did not reach statistical significance (Apr/20 = 1,891; mean = 4,830). In contrast, pediatric visits exhibited a more profound and sustained disruption. Our data suggests that pediatric ED utilization in 2018 and 2019 followed the established seasonal patterns of respiratory viral circulation in Brazil, which typically peaks from April to June [24]. However, this seasonality was markedly attenuated after 2020. Although not statistically significant, the trough in 2020 represented a 77.9% reduction compared with the baseline mean (Apr/20 = 220; mean = 996); meanwhile, other pediatric centers reported reductions between 20% and 30% [8,25]. Although reductions in pediatric ED visits during the pandemic have been reported elsewhere, the magnitude and persistence observed in our cohort suggest a durable alteration in pediatric care-seeking behavior, potentially driven by prolonged changes in viral epidemiology, parental risk perception, and access to outpatient pediatric services.

Figures 4-7 illustrate the temporal trends in ED visits stratified by medical specialties, revealing substantial heterogeneity in post-pandemic recovery patterns. Internal Medicine (Figure 4) experienced an abrupt decline coinciding with the onset of the pandemic, followed by partial recovery and subsequent fluctuations, which could be related to heightened avoidance of hospital settings during periods of restricted mobility, particularly for symptoms perceived as non-urgent, and possible sustained behavioral change in care-seeking, such as telemedicine and delayed ED use. In contrast, neurology visits (Figure 5) demonstrated a sustained upward trajectory throughout the entire study period, with a significantly positive slope even during the pandemic years, which could be possibly explained by factors like heightened reliance on tertiary emergency services for time-sensitive neurologic complaints or disruptions in outpatient follow-up and primary care access that may have redirected neurologic patients toward EDs. Psychiatry (Figure 6) largely mirrored overall hospital trends, showing an abrupt reduction at the start of the pandemic, although it was not statistically significant, followed by gradual stabilization at lower levels compared with the pre-pandemic baseline. Ophthalmology visits (Figure 7) showed a declining trend beginning prior to the pandemic, with an abrupt increase from May 2020 to July 2021, followed by a brief rebound in late 2021, and then stabilized at markedly reduced volumes. This pattern is consistent with the substantial contraction of elective and outpatient ophthalmologic services reported at our institution during the pandemic period [26].

Figures 8-10 depict the temporal evolution of ED visits across surgical specialties, again highlighting divergent recovery trajectories. Trauma and emergency surgery (Figure 8) demonstrated marked variability over time, with an initial drop during the pandemic period, followed by a pronounced rebound and subsequent sustained growth, eventually surpassing pre-pandemic levels. This late-stage growth may reflect the resumption of high-risk activities following the relaxation of mobility restrictions and possible changes in referral patterns favoring tertiary trauma centers during the post-pandemic period.

Neurosurgery visits (Figure 9) showed a modest increase in the early years of the study, but this trend changed after 2020, acquiring a slowly decreasing pattern and stabilizing at lower volumes than expected from the pre-pandemic peak. This shift could be related to prioritization policies favoring urgent over elective neurosurgical cases, as well as prolonged disruptions in diagnostic pathways and referral flows. Orthopedic visits (Figure 10) showed substantial variability, with a sharp but non-significant decline in 2020 and persistent post-pandemic volumes remaining below baseline. Our findings about the surgical specialties in our ED seem to be mostly consonant with those regarding the reduction in the number of elective surgeries in HC-UNICAMP [16].

This study has important limitations. First, it is a single-center analysis. Despite serving a large and diverse macro-regional population, our findings may not generalize to settings with different structures, referral dynamics, or resource availability. Second, the study relies solely on electronic medical record logs, which, although prospectively maintained by the hospital’s informatics team, remain vulnerable to miscoding and incomplete specialty classification that the authors could not independently verify. Third, the dataset includes only visit counts, preventing assessment of illness severity, admission patterns, return visits, or outcomes, thereby limiting the possibility of inferences about the drivers of changing ED demand. In addition, temporal trends may have been influenced by unmeasured confounding factors, including shifts in referral patterns, primary care access, socioeconomic disruptions, population behavior, and institutional policies implemented during and after the pandemic. Finally, the retrospective design and application of joinpoint regression allow identification of temporal inflection points but cannot establish causality with respect to public health measures, viral transmission patterns, or individual care-seeking behavior.

Moreover, the categorization of pre-pandemic, pandemic, and post-pandemic periods in this study was based on declarations by the WHO because they were also used by local health authorities and our hospital’s administration to guide public health policies and healthcare organizations. Nevertheless, we acknowledge that the post-pandemic period does not necessarily represent a return to pre-COVID-19 conditions, as ongoing viral circulation, healthcare system inertia, and sustained behavioral changes may continue to influence ED utilization, as our data seem to suggest. Therefore, alternative temporal cut-points, such as vaccination coverage, local incidence rates, or policy changes, could yield different interpretations and warrant exploration in future studies.

## Conclusions

In conclusion, the COVID-19 pandemic was associated with a marked and prolonged disruption in emergency department utilization at our tertiary referral center. Although visit volumes partially recovered over time, they did not fully return to pre-pandemic levels, and distinct, specialty-specific patterns emerged. These findings suggest that the pandemic not only caused transient reductions in emergency care use but may have reshaped patterns of ED demand in a sustained manner. From a policy perspective, these results support the need for flexible, specialty-specific resource planning within emergency departments, as well as continuous monitoring of utilization trends to identify services at risk of persistent underuse or overcapacity. Such adaptive planning may help health systems better align workforce allocation and care pathways in the post-pandemic period and improve preparedness for future public health emergencies.
